# Comparing the SARS-CoV-2-specific antibody response in human milk after homologous and heterologous booster vaccinations

**DOI:** 10.1038/s42003-023-04455-4

**Published:** 2023-01-25

**Authors:** Sien J. Mulleners, Hannah G. Juncker, Eliza J. M. Ruhé, Aniko Korosi, Johannes B. van Goudoever, Marit J. van Gils, Britt J. van Keulen

**Affiliations:** 1 Emma Children’s Hospital, Amsterdam Reproduction & Development Research Institute, Amsterdam University Medical Center (location VUmc), De Boelelaan 1118, 1081 HZ Amsterdam, The Netherlands; 2 Swammerdam Institute for Life Sciences, Science Park 904, 1098 XH Amsterdam, The Netherlands; 3 Amsterdam Infection and Immunity Institute, Amsterdam University Medical Center (location AMC), Meibergdreef 9, 1105 AZ Amsterdam, The Netherlands

**Keywords:** RNA vaccines, Paediatric research, Mucosal immunology

## Abstract

Human milk contains SARS-CoV-2-specific antibodies after COVID-19 vaccination. These milk antibodies decrease several months post-vaccination. Whether booster immunization restores human milk antibody levels, potentially offering prolonged passive immunity for the infant, remains unknown. In this prospective follow-up study, we investigated the longitudinal SARS-CoV-2-specific antibody response in human milk of 26 lactating women who received a COVID-19 booster dose of an mRNA-based vaccine. Moreover, we evaluated whether the booster-induced human milk antibody response differs for participants who received a similar or different vaccine type in their primary vaccination series. All participants (100%) who received a homologous booster vaccination showed SARS-CoV-2-specific immunoglobulin A (IgA) and immunoglobulin G (IgG) in their milk. Heterologous booster vaccination resulted in milk conversion for 9 (69%) and 13 (100%) participants for IgA and IgG respectively. Findings of this study indicate that both homologous and heterologous boosting schedules have the potential to enhance SARS-CoV-2-specific IgA and IgG in human milk.

## Abstract (web summary)

The longitudinal SARS-CoV-2-specific antibody response in human milk of lactating women receiving an mRNA-based booster vaccine indicates that both homologous and heterologous boosters enhance SARS-CoV-2-specific antibodies in human milk.

## Introduction

Infants account for the highest proportion of hospitalized children due to severe coronavirus disease 2019 (COVID-19)^1,2^. In the first months of life, infants mainly rely on passive immunity derived from their mother to protect them from respiratory infections^3^, including infection with the severe acute respiratory syndrome coronavirus-2 (SARS-CoV-2). Human milk offers passive immunity to the breastfed infant through the transfer of disease-specific antibodies, mostly immunoglobulin A (IgA)^4^.

Maternal vaccination against COVID-19 can induce neutralizing SARS-CoV-2-specific human milk antibodies, which likely offer protection to the breastfed infant^5,6^. Vaccine-induced antibodies against SARS-CoV-2 in serum wane after several months, consequently leading to the occurrence of breakthrough infections^7^. A similar decrease was observed for specific human milk antibodies^6^, which may also entail waning protection for the breastfed infant. Booster vaccination can lead to restored immune protection against SARS-CoV-2 by boosting neutralizing antibody titers in serum^8^. Whether booster immunization also leads to restored human milk antibody levels, potentially offering prolonged passive immunity for the infant, remains unknown.

In this prospective cohort study, we demonstrate the longitudinal SARS-CoV-2-specific antibody response in human milk of lactating women receiving a COVID-19 booster dose of an mRNA-based vaccine. These findings indicate that both homologous and heterologous boosting schedules enhance SARS-CoV-2-specific IgA and IgG in milk of lactating women.

## Results

**Fig. 1:**
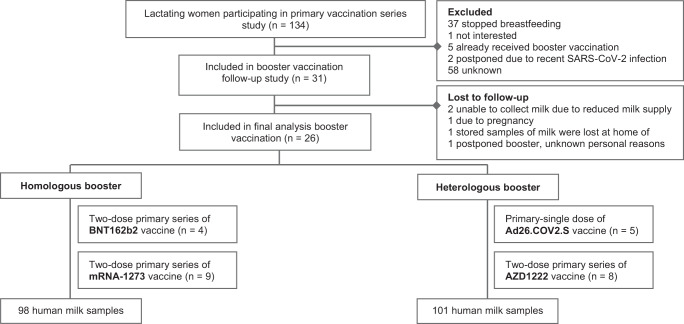
Study flowchart. Flowchart demonstrating the inclusion process of participants in the current study.

Of the 26 lactating women participating in this follow-up study, 13 (50%) received homologous booster vaccination after completing a COVID-19 vaccination regimen with an mRNA-based vaccine, namely BNT162b2 (*n* = 4) or mRNA-1273 (*n* = 9). The other 13 participants (50%) received heterologous booster vaccination after they had previously completed a vaccination regimen with a vector-based vaccine, namely AZD1222 (*n* = 8) or Ad26.COV2.S (*n* = 5). For the booster dose, all participants received the BNT162b2 vaccine, except for one participant who received a booster dose of the mRNA-1273 vaccine (Fig. 1).

Characteristics of participants prior to the first COVID-19 vaccination are shown in Table 1. At the start of the initial study, before vaccination, participants had been breastfeeding for a mean time of 6.6 months (SD, 4.5), and this was similar between the two groups. None of the participants reported a PCR-confirmed SARS-CoV-2 infection prior to vaccination. Participants with specific antibodies targeting the spike protein of SARS-CoV-2 in their serum prior to their first vaccination were excluded from the analysis.

**Table 1:** Cohort characteristics prior to vaccination.

	Homologous (*n* = 12^a^)	Heterologous (*n* = 13)
*Maternal characteristics (N* *=* *26)*		
Age, mean (SD), years	33 (5)	32 (4)
BMI, mean (SD)	24.4 (4.6)	23.7 (3.2)
Chronic disease, no. (%)	2 (16.7)	1 (7.7)
Autoimmune disease, no. (%)	2 (16.7)	0 (0)
Medication use, no. (%)	3 (25)^b^	4 (30.8)^b^
Primiparous, no. (%)	5 (41.7)	7 (58.3)
Vaccination history, no. (%)		
Child immunization	12 (100)	13 (100)
Pertussis vaccine during pregnancy	12 (100)	13 (100)
Other^c^	7 (58.3)	9 (69.2)
Lactation duration, mean (SD), months	5.9 (3.5)	7.2 (5.2)
Nationality parents, no. (%), Dutch	11 (91.7)^d^	13 (100)
*Infant characteristics (N* *=* *26)*		
Sex, no. (%)		
Male	5 (41.7)	7 (53.8)
Female	7 (58.3)	6 (46.2)
Birth weight, mean (SD), g	3545 (583)	3339 (499)
Gestational age, mean (SD), weeks	39.5 (1.5)	39.6 (1.5)
Exclusive breastfeeding, no (%)	8 (66.7)	6 (46.2)

Characteristics at baseline of lactating women included in this follow-up study and of their infants. Items below the bolded heading “Maternal” describe the characteristics of lactating women participating in the current study, and items below the bolded heading “Infant” describe the characteristics of the participants’ infants.

*BMI* body mass index (calculated as weight in kilograms divided by height in meters squared).

^a^One participant in the homologous booster vaccination group did not fill out the questionnaire.

^b^None of the participants took immune system-influencing medication.

^c^Other included vaccinations like travel- or work-related vaccination.

^d^One participant’s father has German nationality.

The mean time to booster vaccination after the first vaccine dose was 285 days (full range, 230–366 days) in the homologous group and 266 days (full range, 214–319 days) in the heterologous group. In total, 199 human milk samples were analyzed.

**Fig. 2:**
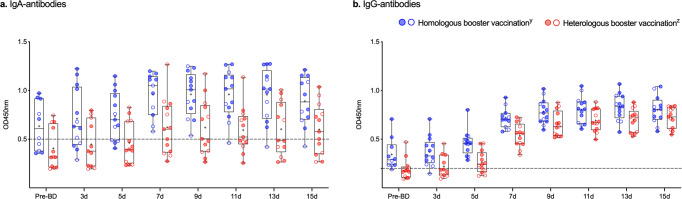
SARS-CoV-2-specific antibodies in human milk after COVID-19 booster vaccination in lactating women. Antibody responses in human milk before and after homologous and heterologous booster vaccination are shown in box-and-whisker plots of SARS-CoV-2-specific immunoglobulin A (**a**) and immunoglobulin G (**b**). Blue dots (y) represent participants who received an mRNA-based vaccine for their primary series: two doses of mRNA-1273 (blue-filled dots) or BNT162b2 (transparent dots, blue border). Red dots (z) represent participants who received an adenoviral vector-based vaccine as primary series: two doses of AZD1222 (red-filled dots) or a single dose of Ad26.COV2.S (transparent dots, red border). For each group, *n* = 13 biologically independent samples except for the following days in the heterologous booster group prior to booster dose (*n* = 12) and after 3 days (*n* = 11), and in the homologous booster group prior to booster dose (*n* = 10), after 7 days (*n* = 11), and 15 days (*n* = 12). All scatter plots in this figure represent the mean of measurements in duplicate per participant. Analyzed samples were collected before (Pre-BD), and 3 days (3d), 5 days (5d), 7 days (7d), 9 days (9d), 11 days (11d), 13 days (13d), and 15 days (15d) after administration of an mRNA-vaccine booster dose (BD). Boxes indicate the 25th percentile, median (horizontal bar), mean (“+”), and 75th percentile; whiskers, minimum and maximum ranges; OD450nm, optical density 450 nm. Cutoff values are indicated with dashed lines. Source data are provided in the Supplementary Data 1 file.

Prior to the administration of the booster dose, mean IgA antibodies in milk were similar for both groups (95% CI of mean 0.08–0.54; *p* = 0.25, *n* = 22 samples). All participants who received homologous booster vaccination showed detectable levels of SARS-CoV-2-specific IgA in their milk at one time point at least (Fig. 2a). Detectable IgA was sustained in the milk of 12 out of 13 participants until the end of the follow-up period. In the heterologous booster vaccination group, 9 out of 13 participants had detectable IgA in their milk during the follow-up period at one time point at least. All participants (100%) had detectable levels of SARS-CoV-2-specific IgG after both homologous and heterologous booster vaccination (Fig. 2b and Supplementary Data 1).

There was no difference in the elicited antibody response following booster vaccination between participants who received AZD1222 or Ad26.COV2.S as a vector-based vaccine for their primary vaccine series.

**Fig. 3:**
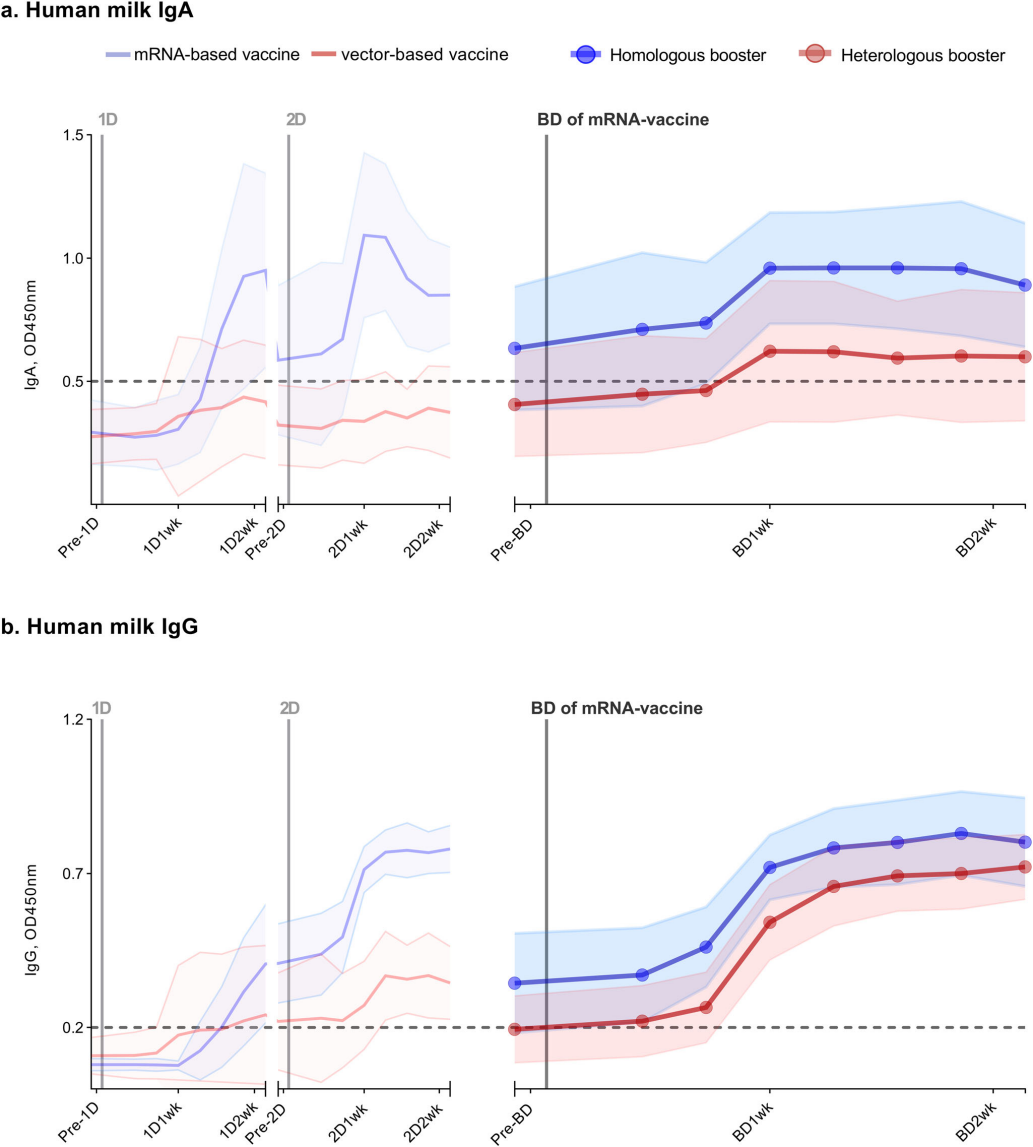
COVID-19 vaccine-induced antibody response in human milk. Antibody response in human milk of 26 lactating women vaccinated against COVID-19. SARS-CoV-2-specific immunoglobulin A (**a**) and immunoglobulin G (**b**) in human milk were measured using an ELISA with the SARS-CoV-2 spike protein. Participants either received a primary series with an mRNA vaccine (2 × mRNA-1273 or 2 × BNT162b2), subsequently following a homologous boosting schedule (*n* = 13), or a vector-based vaccine (2 × AZD1222 or 1 × Ad26.COV2.S) and thus following a heterologous boosting schedule (*n* = 13). The left part of both graphs (from pre-1D to 2D2wk), is already described in our previous study^10^. The time between the first dose and booster dose in the homologous group was, on average, 338 days (BNT162b2) and 262 days (mRNA-1273), and in the heterologous group, 234 days (Ad26.COV2.S) and 285 days (AZD1222). Analyzed samples were collected prior to a vaccine dose, 3 days, 5 days, 7 days, 9 days, 11 days, 13 days, and 15 days after a vaccine dose. 1D, first vaccine dose; 2D, second vaccine dose; BD, mRNA-vaccine booster vaccine; Pre, one day prior to; 1wk, one week after; 2wk, two weeks after vaccine dose. Data points represent the mean of all measurements per group; the shaded area around the mean represents the standard deviation. Cutoff values are indicated with dashed lines. OD450nm, optical density 450 nm. Source data are provided in the Supplementary Data 1 file.

Homologous and heterologous booster vaccination induced a similar change in IgA and IgG antibodies in human milk during the follow-up period, defined as AUC_I_ (Fig. 3, Supplementary Data 1, and Table 2). Participants who received homologous booster vaccination had overall higher levels of SARS-CoV-2-specific IgA and IgG in their human milk (AUC_G_, AUC_PP_, and AUC_Net_) than those who received heterologous booster vaccination, due to overall higher pre-booster levels.

**Table 2:** Comparing the SARS-CoV-2-specific antibody response in human milk following booster vaccination with an mRNA-based vaccine.

	Homologous *N* = 13		Heterologous *N* = 13		Mean difference of AUC		
	df	Mean ± SEM	df	Mean ± SEM	*p* value	*t*-score	95% CI
*Immunoglobulin A*							
AUC_PP_	91	5.44 ± 1.18	94	0.95 ± 0.78	0.002	3.18	−7.27 to −1.71
AUC_Net_	91	5.44 ± 1.18	94	0.55 ± 1.06	0.002	3.08	−8.01 to −1.76
AUC_G_	91	13.44 ± 1.18	94	8.55 ± 1.10	0.003	3.04	−8.06 to −1.71
AUC_I_	91	3.29 ± 1.18	94	2.06 ± 1.10	0.447	0.76	−4.40 to 1.95
*Immunoglobulin G*							
AUC_PP_	91	6.59 ± 0.64	94	4.28 ± 0.45	0.003	2.98	−3.83 to −0.78
AUC_Net_	91	6.59 ± 0.64	94	4.28 ± 0.45	0.003	2.97	−3.83 to −0.77
AUC_G_	91	9.79 ± 0.64	94	7.48 ± 0.52	0.005	2.82	−3.92 to −0.69
AUC_I_	91	4.28 ± 0.64	94	4.38 ± 0.52	0.910	0.11	−1.52 to 1.71

This table shows the areas under the antibody response curve of both study groups, presented as mean with standard errors of the mean as described in ref. ^20^. Values below the bolded heading “Homologous” shows the mean of different areas under the curve (AUC) following homologous booster vaccination, and below “Heterologous” shows those following heterologous booster vaccination. The number following ± indicates the standard error of the mean; AUC_G_, the area under the curve with respect to ground; AUC_I_, with respect to increase; AUC_PP_, with respect to the cutoff, the area of positive peaks; AUC_Net_, with respect to the cutoff, net area which subtracts the area of peaks below the cutoff. GraphPad Prism version 9.1.0 for macOS was used to determine and compare the AUCs. Study groups were compared using an unpaired-sampled *t*-test, with a significance level set at a *p* value <0.05. Below the bolded heading “Mean difference of AUC,” the output of the statistical tests are presented showing the significance level, the *t*-score, and the 95% confidence interval of the mean difference of AUC values between the homologous and heterologous group.

Both mean IgA and IgG antibodies detected in human milk increased significantly after 15 days following vaccination. However, mean IgA levels in milk stagnated between 7 and 15 days following vaccination, even showing a moderate decline, while a gradual increase in mean IgG antibodies in milk was observed from pre-booster to day 7 and day 7 to day 15.

## Discussion

Booster doses against COVID-19 are currently being administered because of the decline in efficacy of the primary vaccination series due to the waning of SARS-CoV-2-specific antibodies^9^. Consistent with findings in serum, a decline in human milk antibodies several months after a primary COVID-19 vaccination was observed^10^. In the current study, after booster vaccination with an mRNA-based vaccine, an increase in SARS-CoV-2-specific antibodies in human milk was observed. Interestingly, booster vaccination led to a comparable increase in human milk antibodies from participants who received a primary series of an mRNA-based vaccine (homologous booster vaccination group) to those who completed a primary series with a vector-based vaccine (heterologous booster vaccination group). However, because participants in the homologous booster vaccination group started from higher pre-booster levels, the total amount of SARS-CoV-2-specific antibodies in their milk over the study period after booster vaccination was higher.

We observed a steady increase in human milk IgG after booster vaccination, while the IgA response was moderate and of greater inter-individual variation. This difference could be explained by the fact that COVID-19 vaccines are intradermally administered, mostly triggering a systemic immune response (IgG).

In addition, IgG appeared earlier than IgA in human milk after booster vaccination, and IgG continued to increase steadily, whereas IgA showed a moderate increase which stagnated after only several days and started to decline thereafter. The diverse dynamics of IgA and IgG responses could be attributed to their different immune functions. IgA is the principal antibody in mucosal immunity and serves as the first line of defense, whereas IgG, the most abundant antibody type in serum, predominantly plays a role during the secondary immune response and is maintained for several months^11^. Similar observations have been described by other researchers studying the humoral response to natural SARS-CoV-2 infection and to different COVID-19 vaccines^12,13^.

Currently, trials supporting recommendations for booster vaccinations in lactating women are lacking. In one study, an increase in specific IgG and IgA levels in milk was observed after administration of a BNT162b2 booster vaccination in 12 lactating women who had received a primary series of BNT162b2^14^. Moreover, heterologous vaccination schedules in lactating women have not been studied, even though many lactating women received heterologous booster vaccination because vector-based vaccines for women below 60 years of age were no longer used in the Netherlands due to reports of severe thrombosis. Our study adds that the administration of an mRNA-based booster vaccination induces a significant antibody response in human milk independent of the initial vaccine type.

Although previous data on human milk is lacking, findings from a randomized controlled trial also show that both homologous and heterologous booster vaccinations induce a sufficient systemic antibody response and that these booster-induced antibodies in serum have the capacity to neutralize SARS-CoV-2^15^.

Timing of sampling and freeze-thaw cycles are suggested to affect human milk composition^16^, but it is unclear whether this also affects IgA. Human milk antibodies have been found to be relatively stable during freeze-thaw cycles, though contradictory observations have been reported^17^. In the current study, all of the collected samples of milk were thawed once. Despite clear instructions to participants on milk sample collection, personal variation in collection and time between collection and storage in the freezer cannot be ruled out since there was no supervision during sample collection. Also, we cannot completely rule out a SARS-CoV-2 infection in between vaccine doses as we did not verify the absence of anti-nucleocapsid antibodies; only the absence of anti-spike protein antibodies was verified prior to vaccination. Therefore, we cannot draw conclusions from comparisons on an individual level, but solely on a group level. Interpretation of our results may also be limited due to our small sample size. But as we only included participants who had already participated in our previous study, this allowed us to demonstrate the longitudinal antibody response following their primary series and booster vaccination. In the current study, we did not collect infant samples. Transmission of vaccine-induced antibodies against SARS-CoV-2 to the breastfed infant has been previously described^18^. Large sample-sized studies are needed to confirm the transfer and immunological protection of human milk antibodies for breastfed infants against SARS-CoV-2 infection.

In summary, our current findings demonstrate the potential of mRNA-based booster vaccination to enhance SARS-CoV-2-specific immunoglobulin A and immunoglobulin G in human milk. Based on our results, we support recommendations on mRNA-vaccine booster doses in lactating women and suggest that both homologous and heterologous boosting induce specific antibodies against SARS-CoV-2 in human milk, which likely protect the breastfed infant.

## Methods

### Study design

To investigate the longitudinal antibody response against SARS-CoV-2 in human milk after COVID-19 booster vaccination, we conducted a prospective cohort follow-up study of previously fully vaccinated individuals who participated in our original vaccination study^10^.

To assess whether the human milk antibody response against COVID-19 differs after homologous and heterologous booster vaccination, participants were divided into two cohorts based on the type of vaccine they received for their primary vaccination series, and human milk samples from both groups were collected longitudinally following booster vaccination.

### Ethical considerations

This study was conducted in accordance with the declaration of Helsinki and the ICH GCP Guidelines and complied with the Regulation on Medical Research Involving Human Subjects. The study protocol was approved by the Ethics Committee of the Amsterdam University Medical Center. We have obtained written informed consent from all participants.

### Population and setting

The current study is a follow-up of our previous vaccination study^10^. All lactating individuals in the Netherlands receiving a vaccination against COVID-19 were eligible to participate. Participants of the initial vaccination study who were still lactating and were going to receive a booster dose of a COVID-19 vaccine could register to participate in the current prospective follow-up study.

Recruitment of participants for the original study was done through several social media platforms and registration for participation in the current follow-up study was requested from participants who had consented to be contacted for possible further research.

### Study groups

The first study group consisted of participants who received an mRNA-based vaccine, namely BNT162b2 or mRNA-1273, both for their primary vaccination series and for their booster vaccination (homologous booster vaccination group). The second study group consisted of lactating women who completed a primary series of an adenoviral vector-based COVID-19 vaccine which included either two doses of  the AZD1222/Vaxzevria vaccine or a single dose of the Ad26.COV2.S vaccine, and for their booster vaccination, they received an mRNA-based vaccine (heterologous booster vaccination group).

### Sample collection

Over a period of 16 days, participants collected eight human milk samples: one sample before and a sample on days 3, 5, 7, 9, 11, 13, and 15 after booster vaccination. All samples were collected in a standardized way. Participants were instructed to empty one breast completely before the first feeding moment, either manually or with an electric breast pump, mix the milk (so that fore- and hindmilk were mixed), and subsequently store 5 ml in the freezer until collection by one of the researchers during the home visit at the end of the follow-up period. Human milk samples were collected in sterile polypropylene tubes. During transport to the study site, milk samples were placed on dry ice and at the study site, samples were stored at −80 °C up until analysis.

### Antibody analysis in human milk

SARS-CoV-2-specific IgA and IgG antibodies in human milk were determined using an enzyme-linked immunosorbent assay with the SARS-CoV-2 spike protein. A more detailed description was also reported in our previous study^19^ as follows:

Soluble prefusion-stabilized S-protein of SARS-CoV-2 was generated. This protein was immobilized on a 96-well plate (Greiner, Kremsmünster, Austria) at 5 µg/mL in 0.1 M NaHCO_3_ overnight, followed by a 1-h blocking step with 1% casein PBS (Thermo Scientific, Waltham, MA, USA). Human milk was diluted 1:5 in 1% casein PBS and incubated on the S-protein coated plates for 2 h to allow binding. Antibody binding was measured in the human milk samples using 1:3000 diluted HRP-labeled goat anti-human IgG (Jackson Immunoresearch, West Grove, PA, USA) and 1:3000 diluted HRP-labeled goat anti-human IgA (Biolegend, San Diego, CA, USA) in casein. Healthy controls were used (of our previous study) to determine cutoff values defined as the mean plus two times the standard deviation. Sensitivity was 68% for IgA and 96% for IgG in human milk. Specificity was 99% for both IgA and IgG in human milk. All samples of human milk were analyzed as one batch at the same time. Milk samples were considered positive at an optical density 450-nm cutoff value of 0.5 for IgA in human milk (sensitivity, 68%; specificity, 99%) and 0.2 for IgG in human milk (sensitivity, 96%; specificity, 99%). All biologically independent samples were assayed in duplicate.

### Statistics and reproducibility

Characteristics of participants and their infants were compared between study groups, IBM SPSS Statistics for macOS (Version 29) was used. Discrete variables are denoted by the observed number and percentages within a study group, and continuous variables are presented by mean and standard deviation after testing for normality.

Human milk antibody dynamics following COVID-19 vaccination are displayed using Graphpad Prism 9.1.0 for macOS. To assess the total antibody response after booster vaccination, we determined the area under the curve with respect to ground (AUC_G_), with respect to increase from mean pre-booster level (AUC_I_), and the area under the curve with respect to the cutoff: both the area of positive peaks (AUC_PP_) and the net area which subtracts the area of peaks below the cutoff (AUC_Net_), as described in ref. ^20^. The AUCs were calculated for the human milk antibody response up to 15 days after the administration of the booster dose. The mean AUCs with standard errors of the mean (SEM) and degrees of freedom (df) for both study groups were compared using an unpaired-sampled *t*-test, with a significance level set at a *p* value <0.05. GraphPad Prism version 9.1.0 for macOS was used to determine and compare the AUCs.

### Reporting summary

Further information on research design is available in the Nature Portfolio Reporting Summary linked to this article.

## Supplementary information

Description of Additional Supplementary Files

Supplementary Data 1

Reporting Summary

## Data Availability

Data supporting the findings of this study are included in Supplementary Data 1.
