# Supplementary material for: Comparing the SARS-CoV-2-specific antibody response in human milk after homologous and heterologous booster vaccinations
Source: Commun Biol. 2023 Jan 25;6:100. doi: 10.1038/s42003-023-04455-4 (PMC9875178; doi:10.1038/s42003-023-04455-4)
Supplement: Supplementary file 1 — Description of Additional Supplementary Files [file 42003_2023_4455_MOESM1_ESM.pdf]

Description of Additional Supplementary Files

File name: Supplementary Data 1

Description: Source data behind the graphs in the paper (figure 2 and 3).
