# Supplementary material for: Comparing the SARS-CoV-2-specific antibody response in human milk after homologous and heterologous booster vaccinations
Source: Commun Biol. 2023 Jan 25;6:100. doi: 10.1038/s42003-023-04455-4 (PMC9875178; doi:10.1038/s42003-023-04455-4)
Supplement: Supplementary file 3 — Reporting Summary [file 42003_2023_4455_MOESM3_ESM.pdf]

nature portfolio

Corresponding author(s): J.B. van Goudoever

Last updated by author(s): Dec 26, 2022

Reporting Summary

Nature Portfolio wishes to improve the reproducibility of the work that we publish. This form provides structure for consistency and transparency in reporting. For further information on Nature Portfolio policies, see our Editorial Policies and the Editorial Policy Checklist.

Statistics

For all statistical analyses, confirm that the following items are present in the figure legend, table legend, main text, or Methods section.

n/a	Confirmed	
☐	☒	The exact sample size (*n*) for each experimental group/condition, given as a discrete number and unit of measurement
☐	☒	A statement on whether measurements were taken from distinct samples or whether the same sample was measured repeatedly
☐	☒	The statistical test(s) used AND whether they are one- or two-sided<br>*Only common tests should be described solely by name; describe more complex techniques in the Methods section.*
☒	☐	A description of all covariates tested
☐	☒	A description of any assumptions or corrections, such as tests of normality and adjustment for multiple comparisons
☐	☒	A full description of the statistical parameters including central tendency (e.g. means) or other basic estimates (e.g. regression coefficient) AND variation (e.g. standard deviation) or associated estimates of uncertainty (e.g. confidence intervals)
☐	☒	For null hypothesis testing, the test statistic (e.g. *F*, *t*, *r*) with confidence intervals, effect sizes, degrees of freedom and *P* value noted<br>*Give P values as exact values whenever suitable.*
☒	☐	For Bayesian analysis, information on the choice of priors and Markov chain Monte Carlo settings
☒	☐	For hierarchical and complex designs, identification of the appropriate level for tests and full reporting of outcomes
☐	☒	Estimates of effect sizes (e.g. Cohen's *d*, Pearson's *r*), indicating how they were calculated

Our web collection on statistics for biologists contains articles on many of the points above.

Software and code

Policy information about availability of computer code

Data collection	No custom software was used for data collection.
Data analysis	GraphPad Prism version 9.1.0 for macOS was used to determine and compare the area under the curves of antibodies in human milk following homologous and heterologous booster vaccination.

For manuscripts utilizing custom algorithms or software that are central to the research but not yet described in published literature, software must be made available to editors and reviewers. We strongly encourage code deposition in a community repository (e.g. GitHub). See the Nature Portfolio guidelines for submitting code & software for further information.

Data

Policy information about availability of data

All manuscripts must include a data availability statement. This statement should provide the following information, where applicable:

- Accession codes, unique identifiers, or web links for publicly available datasets
- A description of any restrictions on data availability
- For clinical datasets or third party data, please ensure that the statement adheres to our policy

Data supporting the findings of this study have not been deposited in a repository but source data are available in the Supplementary Data 1 file.

Human research participants

Policy information about studies involving human research participants and Sex and Gender in Research.

Reporting on sex and gender	Participants were reported as female in this study, not self-reported, on the basis of having recently given birth to a child. We have not specifically asked participant whether they identify themselves as female. Thus, we have Participants reported the sex of their breastfed infant in the questionnaire at enrollment of the initial study. Information on infant sex is added to the manuscript since this is relevant for our research question, as it is a known factor affecting human milk composition.
Population characteristics	All lactating individuals in the Netherlands receiving vaccination against COVID-19 were eligible to participate in the original vaccination follow-up study. Participants of the initial vaccination follow-up study who were still lactating and were going to receive a booster dose of a COVID-19 vaccine could register to participate in the current prospective follow-up study.
Recruitment	Recruitment of participants for the original study was done through several social media platforms and registration for participation in the current follow-up study was requested form participants who had consented to be contacted for possible further research.
Ethics oversight	Ethical approval was acquired from the Ethics Committee of the Amsterdam University Medical Centre. Written informed consent was obtained from all participants.

Note that full information on the approval of the study protocol must also be provided in the manuscript.

Field-specific reporting

Please select the one below that is the best fit for your research. If you are not sure, read the appropriate sections before making your selection.

☒ Life sciences ☐ Behavioural & social sciences ☐ Ecological, evolutionary & environmental sciences

For a reference copy of the document with all sections, see nature.com/documents/nr-reporting-summary-flat.pdf

Life sciences study design

All studies must disclose on these points even when the disclosure is negative.

Sample size	A total of 199 samples were analyzed in the current follow-up study. Sample size was not predetermined with any statistical methods. Sample size depended on whether participants of the original vaccination study were still lactating at the time they received a booster vaccination and wether they were willing and available to continu to participate.
Data exclusions	No data, collected for the current booster follow-up study, was excluded. For our previous vaccination follow-upstudy, the participants who had specific antibodies targeting the spike protein of SARS-CoV-2 in their serum before their first vaccine dose were already excluded from analysis.
Replication	All samples were assayed in duplicate as reported in the manuscript.
Randomization	Findings of this study are observational and non-experimental. Participants were not randomized.<br>In this prospective follow-up study, participants were divided in to study groups, based on the type of vaccine they received for their primary series. In the Netherlands, two mRNA-based and two vector-based vaccines were available for a primary COVID-19 vaccine series. Which type of vaccine a participant received was allocated by the government.
Blinding	The investigators were not blinded during data collection and/or analysis.

Reporting for specific materials, systems and methods

We require information from authors about some types of materials, experimental systems and methods used in many studies. Here, indicate whether each material, system or method listed is relevant to your study. If you are not sure if a list item applies to your research, read the appropriate section before selecting a response.

Materials & experimental systems

n/a	Involved in the study
☐	☒ Antibodies
☒	☐ Eukaryotic cell lines
☒	☐ Palaeontology and archaeology
☒	☐ Animals and other organisms
☒	☐ Clinical data
☒	☐ Dual use research of concern

Methods

n/a	Involved in the study
☒	☐ ChIP-seq
☒	☐ Flow cytometry
☒	☐ MRI-based neuroimaging

Antibodies

Antibodies used

HRP-labeled goat anti-human IgA (Biolegend, San Diego, CA, USA) and HRP-labeled goat anti-human IgG (Jackson, Immunoresearch).

Validation

Antibody binding was measured using 1:3000 diluted HRP-labeled goat anti-human IgG (Jackson Immunoresearch, West Grove, PA, USA) and 1:3000 diluted HRP-labeled goat anti-human IgA (Biolegend, San Diego, CA, USA) in casein for the human milk samples. The healthy controls (human milk) were used to determine cut-off values defined as the mean plus two times the standard deviation.
Sensitivity was 68% for IgA and 96% for IgG in human milk.
Specificity was 99% for both IgA and IgG in human milk.
